# Butyric acid alleviated chronic intermittent hypoxia-induced lipid formation and inflammation through up-regulating HuR expression and inactivating AMPK pathways

**DOI:** 10.1042/BSR20203639

**Published:** 2021-06-21

**Authors:** MiaoShang Su, Yifan He, Sichen Xue, Jueke Yu, Xikai Ren, Nan Huang, Rukkaiya Abdullahi, Manhuan Xu

**Affiliations:** 1 The Second Affiliated Hospital and Yuying Children’s Hospital of Wenzhou Medical University, Zhejiang 325000, China; 2 Laboratory Medical and Life Science College, Wenzhou Medical University, Zhejiang 325035, China

**Keywords:** AMPK, butyric acid, chronic intermittent hypoxia, human antigen R, lipid formation

## Abstract

To investigate whether butyric acid could alleviate chronic intermittent hypoxia (CIH)-induced lipid formation in human preadipocytes-subcutaneous (HPA-s) through accumulation of human antigen R (HuR) and inactivation of AMP-activated protein kinase (AMPK) pathway, HPA-s were obtained and divided into three groups: Control group: cells were cultured under normal conditions; CIH group: cells were cultured in a three-gas incubator (10% O_2_); Butyric acid group: 10 mmol/l butyric acid added into cell culture medium. HuR-siRNA was futher transfected into CIH group for verification the function of HuR. Oil Red O was implemented for observation of lipid droplets within cells. Cell Counting Kit-8 (CCK8) assay was used for detecting cell viability. Terminal deoxynucleotidyl transferase-mediated deoxyuridine triphosphate-nick end labeling (TUNEL) assay as well as flow cytometry analysis was employed for determining cell apoptosis. Western blotting was used for measurement of protein expression levels. RT-qPCR analysis was used for detecting mRNA expression. CIH treatment increased adipocytes proliferation, while butyric acid inhibited cell proliferation and promoted cell apoptosis. The treatment of butyric acid in CIH group down-regulated expression of inflammatory factors and increased cell apoptotic rate. Butyric acid treatment increased HuR expression in both cytoplasm and nucleus and decreased the level of p-AMPK and p-ACC, while transfection of AMPK activator or HuR-siRNA would down-regulate HuR expression. Moreover, butyric acid alleviated CIH-induced cell proliferation, lipid formation and inflammatory status and promoted cell apoptosis through regulating related genes including *p21, PPARγ, C/EBPa, IL-1β, IL-6, TLR4, caspase-8* and *caspase-3*. In conclusion, butyric acid could alleviate CIH-induced inflammation, cell proliferation and lipid formation through accumulation of HuR and inactivation of AMPK pathway.

## Introduction

Butyric acid, as one of saturated short-chain fatty acids, which is the main component of fats in the foods and lipids, affords health benefits against lipid disorders [1]. In recent years, obesity, a systemic inflammatory disease, characterized by excessive lipid storage in adipose tissue, has been an increasingly significant public health problem [2,3]. People suffering from obesity have a higher prevalence of metabolic disorders such as cardiovascular disease, diabetes and some types of cancer [4]. In addition, a clinical research indicated that children with obesity had a shorter sleep time and a higher incidence of obstructive sleep apnea [5], which leads to chronic intermittent hypoxia (CIH). Recent evidence suggests that butyric acid is beneficial to the lipid disorders [6] and exerts a dramatic hypotensive effect at a dose [7]. However, little attention has been paid to the mechanism of inhibiting lipogenesis by butyric acid.

As a major repressor during adipogenesis, human antigen R (HuR) plays a critical role in RNA metabolism [8]. And HuR protein is expressed in various cell types, such as adipose, intestine, spleen and testis [9]. Generally, HuR improves the efficiency of translation by positively regulating target mRNAs and their stability [10]. Researches show that HuR is intimately related to the inflammation, apoptosis, proliferation and polarization [11,12] and is a crucial repressor of adipogenesis and negatively regulate the inflammation [8]. Therefore, HuR has been identified to have a potential impact on lipid metabolism and suppress the inflammation ulteriorly. However, mechanism insights are currently lacking. In consideration of the functions of HuR, it is vital and urgent to find how it affects lipogenesis and inflammation. AMP-activated protein kinase (AMPK), as a regulator of metabolism, could inhibit cytoplasmic export of HuR by influencing the mRNA turnover [13], and exert a wide range of benefits on energy homeostasis [14]. Thus, the mechanism of adipogenesis and anti-inflammation is set out to investigate further. Recently investigators have examined the effect of CIH on inducing oxidative stress and cytokines production, which was commonly found in obese suffering from obstructive sleep apnea [15,16]. Surveys such as that conducted by Su et al. have shown that frequent episodes of CIH result in the increase in excitability in bladder receptors, leading to bladder dysfunction, indicating the destructive effects of CIH on physical functions [17].

In the present study, we hypothesized that butyric acid could increase cytoplasm accumulation of HuR and regulate the expression of its downstream genes via inhibiting the activation of AMPK in CIH, thus, promotes apoptosis to inhibit the generation of adipocytes and plays an important role in anti-inflammatory property.

## Methods

### Reagents

Poly-l-lysine medium was purchased from Sigma–Aldrich, St. Louis, MO, U.S.A.; preadipocyte medium (PAM, C-27410), preadipocyte differentiation medium (PADM, C-27436) and adipocyte medium (ADM, C-27438) were obtained from PromoCell, Heidelberg, Germany; Oil Red O kit, annexin V-FITC solution and propidium iodide solution were bought from Solarbio, Beijing, China; Cell Counting Kit-8 (CCK8) was purchased from KeyGen Biotech, Nanjing, China; Terminal deoxynucleotidyl transferase-mediated deoxyuridine triphosphate-nick end labeling (TUNEL) assay was obtained from Vazyme Biotech, Beijing, China. All the antibodies used were purchased from Abcam Co., Ltd., Cambridge, U.S.A.; TRIzol reagent and TaqMan MicroRNA reverse transcription kit were bought from Invitrogen, Waltham, MA, U.S.A. All the primers and siRNAs were synthesized at GenePharma, Shanghai, China.

### Cell culture

Human preadipocytes-subcutaneous (HPA-s) were obtained from ScienCell Research Laboratories, Carlsbad, CA, U.S.A. Cells were inoculated in a poly-l-lysine medium at a density of 1 × 10^5^ cells/cm^2^, and then cultured in an incubator with 5% CO_2_ at 37°C for 2 days using PAM. When the cell fusion degree reached 80%, PAM was replaced by PADM which was refreshed every 3 days. After 7 days, human preadipocytes differentiated into mature adipocytes that were cultured in ADM.

Cells were divided into three groups: Control group: cells were cultured under normal conditions; CIH group: cells were incubated in CIH chamber and exposed to 9-h deoxygenation–reoxygenation cycle of 5% oxygen for 60 min and 20% oxygen for 30 min [18]; Butyric acid group: 10 mmol/l butyric acid added into cell culture medium.

### Oil Red O staining

Oil Red O and distilled water were mixed for 10 min at room temperature in the ratio of 3:2, after which the mixture was filtered. Human preadipocytes and adipocytes which have been induced for 7 days were removed and washed with PBS for three times. The cells were fixed with 4% paraformaldehyde at room temperature for 15 min, and washed with PBS for three times. The cells were stained with Oil Red O for 30 min at room temperature and washed with PBS again. After drying, a microscope was implemented for observation and recording.

### CCK8 assay

Mature adipocytes were collected to prepare cell suspension, after which cells were inoculated into 96-well plates (2 × 10^4^ cells per well), and then cultured in an incubator with 5% CO_2_ at 37°C for 12 h. After implementation of different treatments in each group and incubated for another 48 h, 10 μl CCK-8 solution was added into each well and incubated for 2 h. The absorbance of each well was detected at 450 nm wavelength.

### TUNEL assay

TUNEL assay was obtained for detecting the effect of different treatments on apoptosis of adipocytes. Cells in each group were fixed with 4% paraformaldehyde at room temperature for 15 min and washed with PBS for three times, after which cells were treated with 1% Triton X-100 solution for 3 min and then washed again with PBS. TDT enzyme reaction solution were prepared as follows: 1.0 μl biotin-11-dUTP and 4.0 μl TDT enzyme were added into 45 μl Equilibration Buffer. Then 50 μl of TDT enzyme reaction solution was added to each sample, and incubated in dark at 37°C for 60 min. Streptavidin-fluorescein reagent mixed with 45 μl labeling buffer according to the manufacturer’s instructions and was added into cells and maintained in dark at 37°C for 30 min. 4′-6-Diamidino-2-phenylindole (DAPI) staining solution was used to re-stain the nucleus and incubated in dark 37°C for 10 min. A microscope was used for observation and recording.

### Flow cytometry analysis

The cell culture medium was digested with trypsin at room temperature until the adherent cells were blown down, after which the cells were transferred into a centrifuge tube and centrifuged at 1000×***g*** for 5 min. The supernatant was discarded, and the cells were collected and washed with PBS. Then 195 μl annexin V-FITC solution was added, after which 10 μl propidium iodide solution was added and incubated for 20 min at room temperature (20–25°C). After washing with PBS, cells were resuspended with 300 μl PBS and detected by flow cytometry.

### Western blotting

The cells were placed into a 1.5 ml EP tube and washed twice with PBS. After centrifugation, protein lysate was added and the cells were lysed at 4°C for 1 h. The lysed protein solution was centrifuged at 12000×***g*** for 10 min, and the supernatant was collected and the concentration of protein was measured using a BCA protein assay kit. Total protein was separated with SDS/PAGE and transferred on to a PVDF membrane. The membrane was then incubated in blocking liquid for 1 h at 25°C. After the removal of rest liquid, the membrane was probed by primary antibodies of IL-1β (ab200478, 1:500), IL-6 (ab6672, 1:500), IFN-r (ab 224197, 1:500), TLR4 (ab22048, 1:500), HuR (ab200342, 1:500), AMPK (2532, Cell Signaling, 1:500), p-AMPK (2531, Cell Signaling, 1:500), p-ACC (11818, Cell Signaling, 1:500), p21 (ab109520, 1:500), caspase-8 (ab32397, 1:500), caspase-3 (ab32351, 1:500), cleaved caspase-8 (9748, Cell Signaling, 1:500) cleaved caspase-3 (ab32042, 1:500), PPARγ (ab178860, 1:500), C/EBPa (ab40761, 1:500) and GAPDH (ab8245, 1:1000). The corresponding secondary antibodies were applied together with ECL chromogenic solution. The Western blotting results were quantified by gray value analysis using an Image analyzer quantitative system.

### RT-qPCR analysis

The total RNA was extracted from cells using TRIzol reagent. cDNA was synthesized using the TaqMan MicroRNA reverse transcription kit according to the manufacturer’s instructions with the following condition: 16°C for 30 min, 37°C for 30 min, 70°C for 10 min. The RT-qPCR analysis was performed on CFX96 Real-Time PCR Detection System with SYBR Green Master Mix. The *GAPDH* gene served as the internal reference and other primers were listed in Supplementary Table S1. The relative expression of mRNA was calculated by the formula of 2^−△△*C*_t_^ method.

### HuR-siRNA transfection

Cells were diluted using 10% FBS without antibiotics and then distributed into a six-well plate at a density of 5 × 10^4^/cells, after which cells were incubated in a 5% CO_2_ incubator at 37°C for 24 h. The transfection of HuR-siRNA was performed according to the manufacturer’s instructions. Briefly, 5 μl siRNA and 5 μl Lipo2000 was dissolved into 50 μl serum-free medium and incubated at room temperature for 5 min, respectively. Then the solution was mixed and incubated at room temperature for 20 min and added into cells. Cells were further incubated in a 5% CO_2_ incubator at 37°C for 24 h.

### Statistical analysis

All experiments were performed in triplicates and all statistical data were expressed as ±standard deviation (SD). The comparisons among multiple groups were performed using one-way analysis of variance (ANOVA). If the variance was homogeneous, LSD test was used for further pairwise comparison, else nonparametric test was used and Kruskal–Wallis H test was used for further pairwise comparison via SPSS 24.0. A value of *P*<0.05 was considered statistically significant.

## Results

### Butyric acid treatment increased the apoptosis rate of adipocytes

**Figure 1:**
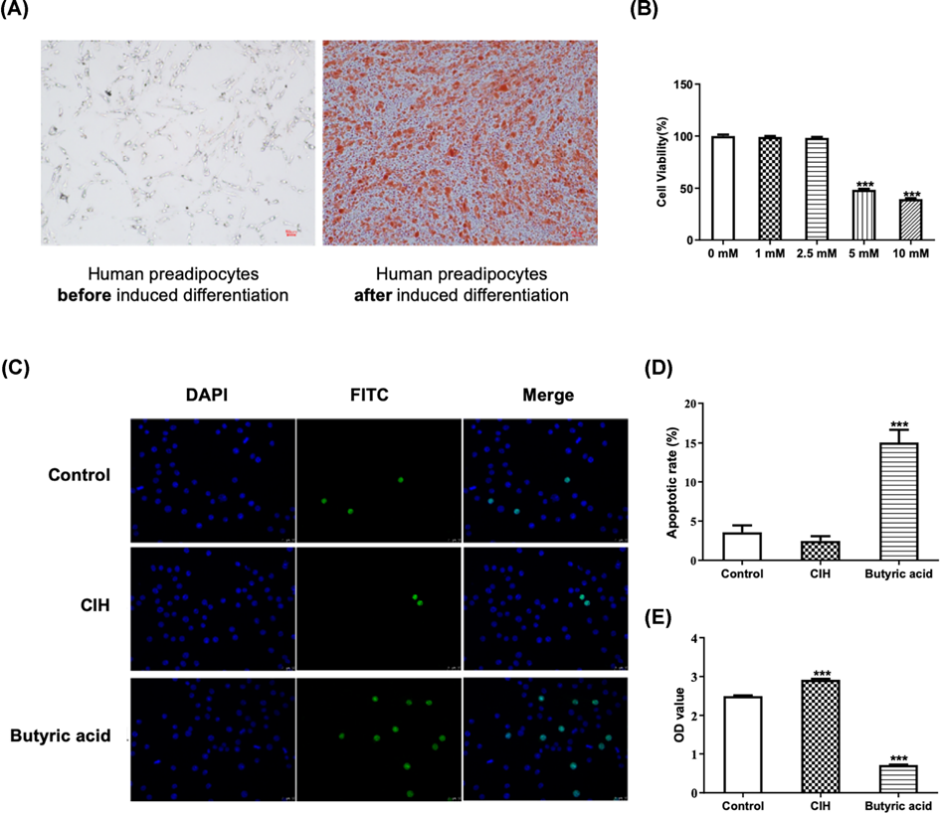
The differentiation of human preadipocytes and effects of CIH and butyric acid on adipocytes Cells in CIH group were treated with 9-h deoxygenation–reoxygenation cycle and butyric acid group received 10 mmol/l butyric acid treatment at day 0, and then followed by adipocyte differentiation for 7 days. (**A**) Oil Red O staining results of human preadipocytes before (left) and after (right) induced differentiation. (**B**) Cell viability under different concentration of butyric acid. (**C**) Cell apoptosis determined using TUNEL assay in control, CIH and butyric acid groups. (**D**) Apoptotic rate in different groups. (**E**) Cell proliferation detected by OD value in different groups. ***P<0.001 vs Control.

HPA-s were cultured in PADM and observed under microscope. After 4 days of culturing, lipid droplets were observed, which were growing into larger droplets with the extension of induction time. After 7 days, the lipid droplets in the cytoplasm were stained using Oil Red O staining (Figure 1A), whose results indicated that HPA-s were successfully differentiated into adipocytes. For determining the appropriate concentration of butyric acid, 1, 2.5, 5 and 10 mmol/l of butyric acid were added into adipocytes, after which cells were cultured at 37°C in a 5% CO_2_ incubator for 24 h. Then cell viability of each group was detected. As presented in Figure 1B, compared with the control group, the cell viability was significantly decreased in butyric acid group when the concentration was 5 and 10 mmol/l (*P*<0.001). Therefore, 2.5 mmol/l of butyric acid was selected for subsequent experiments. After 48 h of hypoxia or butyric acid treatment (Figure 1C,D), no significantly difference in the apoptotic rate were observed in CIH group compared with control group (*P*>0.05). However, the apoptotic rate in butyric acid group was significantly increased (*P*<0.001). The OD value in the CIH group was dramatically increased (Figure 1E), while it was decreased in butyric acid group compared with control group (*P*<0.001). These results suggested that hypoxia treatment had no significant effect on apoptosis and could promote the proliferation of adipocyte, while butyric acid treatment would induce adipocyte apoptosis and suppress cell proliferation.

### Butyric acid alleviated CIH-induced lipid formation, cell proliferation and inflammatory status in adipocytes

**Figure 2:**
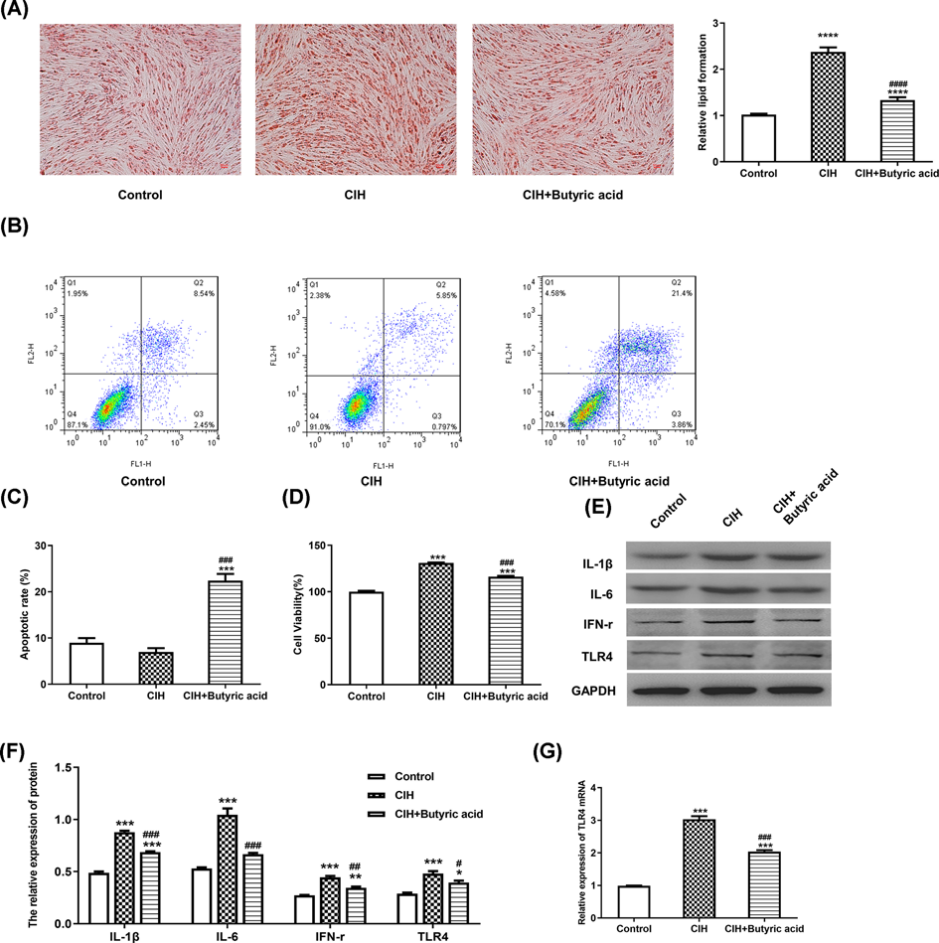
The effects of butyric acid treatment in CIH group Cells in model group were treated with 9-h deoxygenation–reoxygenation cycle and CIH+butyric acid group received 10 mmol/l butyric acid treatment at day 0, and then followed by adipocyte differentiation for 7 days. (**A**) The formation of lipid droplets detected using Oil Red O staining. (**B**) Flow cytometry analysis implemented for measurement of cell apoptosis. (**C**) Apoptotic rate. (**D**) Cell viability determined using CCK8 assay. (**E**) Western blotting results of IL-1β, IL-6, IFN-r and TLR4. (**F**) Relative protein expression calculated via normalization to the protein level of GAPDH. (**G**) The relative mRNA expression of TLR4 determined using RT-qPCR. *P<0.05 vs Control; **P<0.01 vs Control; ***P<0.001 vs Control; ****P<0.0001 vs Control; ^#^*P*<0.05 vs CIH; ^##^*P*<0.01 vs CIH; ^###^*P*<0.001 vs CIH; ^####^*P*<0.0001 vs CIH.

For investigating the effects of butyric acid on CIH-treated adipocytes, Oil Red O staining was used and results showed that the lipid droplets were increased in the CIH group, while addition of butyric acid could reverse the effects (Figure 2A). Flow cytometry analysis was further implemented to determine the cell apoptosis. As shown in Figure 2B,C, the apoptotic rate was significantly increased in CIH + butyric acid group compared with control group and CIH group (*P*<0.001). Cell viability results (Figure 2D) demonstrated that though cell viability was increased in CIH group compared with control group (*P*<0.001), the treatment of butyric acid could decrease the cell viability but could not recover to the level in control group (*P*<0.001). Moreover, inflammation status was evaluated via detection the level of inflammation-related proteins using Western blotting (Figure 2E,F). Data revealed that the expressions of IL-1β, IL-6, IFN-r and TLR4 were up-regulated in CIH group compared with control group, however, these expressions were down-regulated in CIH + butyric acid group compared with CIH group (*P*<0.01). RT-qPCR analysis was employed for determining relative mRNA expression. Figure 2G illustrated that the expression of TLR4 was up-regulated in CIH group compared with control group, while treatment of butyric acid had reverse effect but could not recover to the normal level in control group (*P*<0.001).

### Butyric acid inactivated AMPK pathway and up-regualted HuR expression

**Figure 3:**
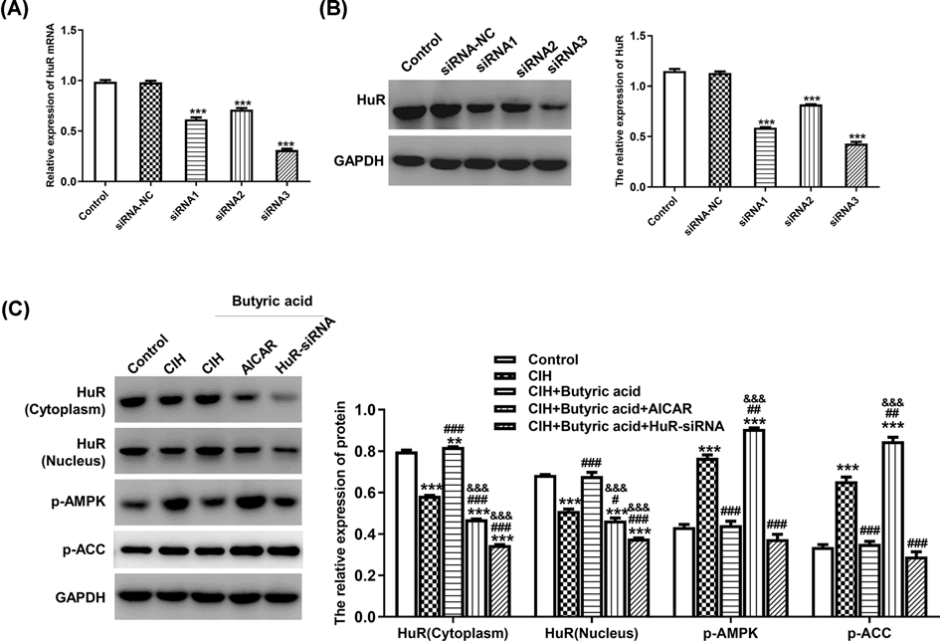
The effects of transfection of HuR-siRNA and AICAR Cells in CIH+butyric acid group were transfected with HuR-siRNA and AICAR at day 0, and then followed by adipocyte differentiation for 7 days. (**A**) The relative mRNA expression of HuR after transfection of HuR-siRNA. (**B**) The protein expression of HuR after transfection of HuR-siRNA determined by Western blotting. (**C**) The protein expression of HuR (cytoplasm), HuR (nucleus), p-AMPK and p-ACC after transfection of AICAR and HuR-siRNA into CIH + butyric acid group. **P<0.01 vs Control; ***P<0.001 vs Control; ^#^*P*<0.05 vs CIH; ^##^*P*<0.01 vs CIH; ^###^*P*<0.001 vs CIH; ^&&&^*P*<0.001 vs CIH + Butyric acid.

In our pre-experiments, it was showed that the level of HuR was significantly decreased under CIH treatment, while butyric acid could up-regulate HuR expression. Therefore, we hypothesized that HuR might be an important regulatory factor in CIH treatment, which was further knocked down using HuR-siRNA to investigate its molecular mechanism. After transfection of HuR-siRNA into CIH group, the mRNA level of HuR was detected using RT-qPCR for efficacy verification. Results presented in Figure 3A indicated that the expression of HuR was down-regulated in siRNA1, siRNA2 and siRNA3 group compared with control group and siRNA-NC group (*P*<0.001). Consistently, the implementation of Western blotting also demonstrated that transfection of HuR-siRNA could down-regulate HuR expression (Figure 3B). Then AICAR, a kind of AMPK pathway activator, was also added into adipocytes. Western blotting results presented in Figure 3C showed that the treatment of butyric acid in CIH group could up-regulate the expression of HuR in both cytoplasm and nucleus and down-regulate the expression of p-AMPK and p-ACC (*P*<0.05). However, the treatment of AICAR and HuR-siRNA could suppress HuR expression, demonstrating that activation of AMPK might lead to the decrease in HuR level.

### HuR suppressed proliferation, lipid formation and inflammatory status in adipocytes

**Figure 4:**
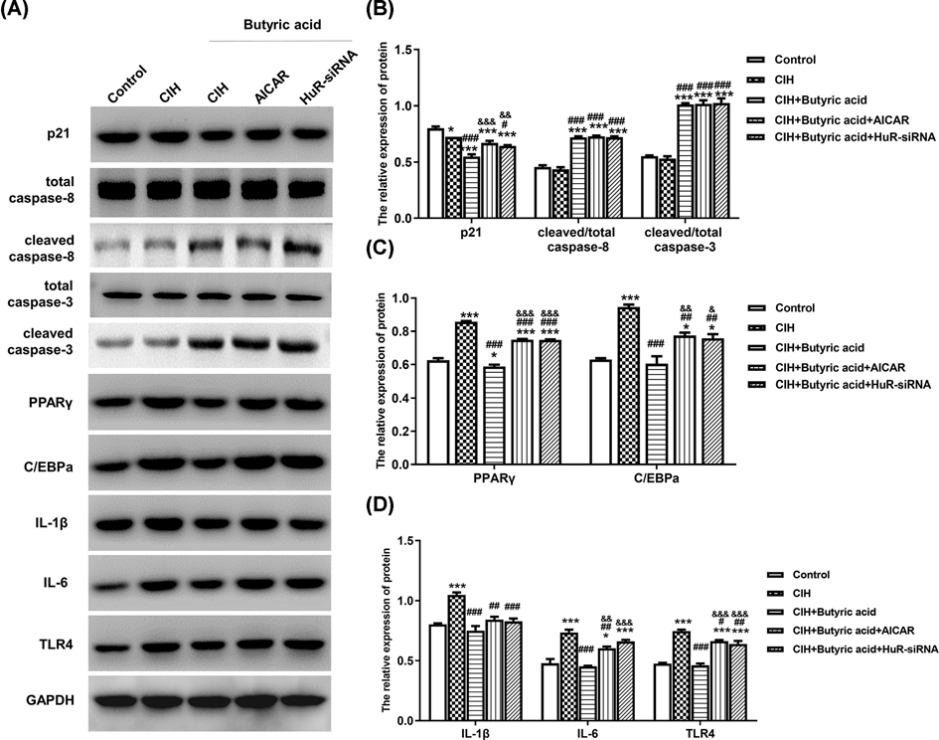
The effects of HuR on proliferation, lipid formation, inflammatory status and apoptosis in adipocytes Cells in CIH+butyric acid group were transfected with HuR-siRNA and AICAR at day 0, and then followed by adipocyte differentiation for 7 days. (**A**) The protein expression of p21, caspase-8, caspase-3, PPARγ, C/EBPa, IL-1β, IL-6 and TLR4. (**B**) The relative protein expression of p21, caspase-8, caspase-3 calculated via normalization to the level of GAPDH. (**C**) The relative protein expression of PPARγ and C/EBPa calculated via normalization to the level of GAPDH. (**D**) The relative protein expression of IL-1β, IL-6 and TLR4 calculated via normalization to the level of GAPDH. *P<0.05 vs Control; **P<0.01 vs Control; ***P<0.001 vs Control; ^#^*P*<0.05 vs CIH; ^##^*P*<0.01 vs CIH; ^###^*P*<0.001 vs CIH; ^&^*P*<0.05 vs CIH + Butyric acid; ^&&^*P*<0.01 vs CIH + Butyric acid; ^&&&^*P*<0.001 vs CIH + Butyric acid.

**Figure 5:**
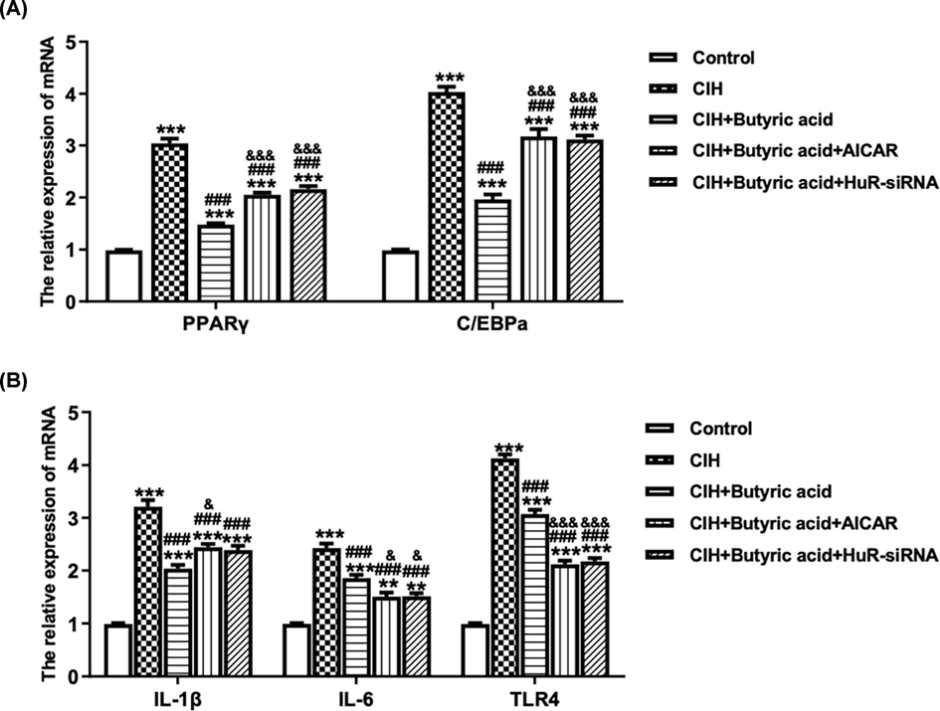
The effects of HuR on lipid formation, inflammatory status Cells in CIH+butyric acid group were transfected with HuR-siRNA and AICAR at day 0, and then followed by adipocyte differentiation for 7 days (**A**) The relative mRNA expression of PPARγ and C/EBPa calculated via normalization to the level of GAPDH. (**B**) The relative mRNA expression of IL-1β, IL-6 and TLR4 calculated via normalization to the level of GAPDH. **P<0.01 vs Control; ***P<0.001 vs Control; ^###^*P*<0.001 vs CIH; ^&^*P*<0.05 vs CIH + Butyric acid; ^&&&^*P*<0.001 vs CIH + Butyric acid.

Western blotting was used to determine the expression of proliferation-related protein (p21), apoptosis-related proteins (caspase-8, caspase-3), lipid formation-related proteins (PPARγ, C/EBPa) and inflammation-related proteins (IL-1β, IL-6, TLR4). As shown in Figure 4A–D, the expression of p21, PPARγ, C/EBPa, IL-1β, IL-6 and TLR4 were down-regulated, while caspase-8 and caspase-3 were up-regulated in CIH + butyric acid group compared with CIH group. However, the activation of AMPK using AICAR or suppressing of HuR using HuR-siRNA had the reverse effect, while no significant change on the relative expression of cleaved caspase-8 and cleaved caspase-3 was observed. Consistently, RT-qPCR results in Figure 5A,B also demonstrated the phenomenon. These evidences suggested that butyric acid could inhibit the formation of lipid and alleviate inflammatory status via regulating the expression of HuR.

## Discussion

Nowadays, metabolic syndrome is a progressively common disease throughout the world [19]. And adipose tissue is responsible for multiple complications induced by obesity, such as breast cancer [20], diabetes [21], gastrointestinal cancers [22] etc. Some studies highlighted that butyric acid played a critical role in lipid metabolism [7]. Ochiai et al. [23] found that butyric acid could induce apoptosis in murine and human T and B cells. Heimann et al. [24] showed butyric acid affected on fat storage and mobilization along with glucose uptake in rat primary adipocytes. In addition, evidence has suggested that CIH contributes to dyslipidemia and arteriosclerosis [25]. Miyako et al. found that chronic inflammation has become a common cause of obesity and adipose tissue remodeling could result in the impairment of adipose tissue function [26]. Thus, exposure to CIH was used to induce lipid formation, cell proliferation and inflammatory status in adipocytes. On the other hand, AMPK is a critical regulator of energy homeostasis regulated by HuR, which also regulate metabolic energy balance [27]. Apart from this, Zhang et al. [28] illustrated that HuR had promising effect on regulating lipid transport and ATP synthesis, indicating the possible pathway for AMPK regulation. Although a large number of researchers have focused on the mechanism of lipid metabolism, the functions of butyric acid in inhibiting adipogenesis and anti-inflammatory mechanism remain elusive and are rarely reported. Thus, various studies have been conducted to explore the specific interaction between butyric acid and HuR.

The present study was designed to determine the effect of HuR, which might be regulated by AMPK. Compared with CIH treatment, butyric acid increased the apoptotic rate of adipocytes. To investigate the effects of butyric acid on CIH-treated adipocytes, Oil Red O staining was used, and inflammation status was evaluated via detection the level of inflammatory-related proteins by western blotting. It was suggested that butyric acid alleviated CIH-induced lipid formation, cell proliferation and inflammatory status in adipocytes. According to the result of Western blotting, the treatment of AICAR and HuR-siRNA could suppress HuR expression, indicating that activation of AMPK might lead to the decrease in HuR level. Next, expression of inflammation-related proteins, HuR, and AMPK were determined by qPCR. It was interesting to note that in the present study butyric acid could inhibit the formation of lipid and alleviate inflammatory status via regulating the expression of HuR. However, the relative expression of cleaved caspase-3 and cleaved caspase-8 was not significantly changed via suppressing HuR or activation of AMPK, indicating that there may be other signaling pathways involved in regulating adipocytes proliferation and apoptosis besides AMPK/HuR. Hence, it could conceivably be hypothesized that butyric acid could result in cytoplasm accumulation of HuR and regulate the expression of its downstream genes via inhibiting the activation of AMPK with exposure to CIH, thus, inhibited the generation of adipocytes and plays an important role in anti-inflammatory property. However, there are still many unanswered questions about the combination between butyric acid and AMPK. Whether butyric acid could regulate AMPK activation through its upstream pathways that included PI3K/Akt pathway or liver kinase B1 would be investigated in future experiments. The specific mechanism of regulation regarding to HuR in RNA metabolism should be illuminated. Therefore, a further study with more focus on the limitation above is therefore suggested.

## Supplementary Material

Supplementary Table S1

## Data Availability

All data generated during the study are available from the corresponding authors on request.

## Abbreviations

ADM: adipocyte medium
AMPK: AMP-activated protein kinase
CCK8: cell counting kit-8
CIH: chronic intermittent hypoxia
HPA-s: human preadipocytes-subcutaneous
HuR: human antigen R
PADM: preadipocyte differentiation medium
PAM: preadipocyte medium
TUNEL: terminal deoxynucleotidyl transferase-mediated deoxyuridine triphosphate-nick end labeling
