# Supplementary material for: Butyric acid alleviated chronic intermittent hypoxia-induced lipid formation and inflammation through up-regulating HuR expression and inactivating AMPK pathways
Source: Biosci Rep. 2021 Jun 21;41(6):BSR20203639. doi: 10.1042/BSR20203639 (PMC8220371; doi:10.1042/BSR20203639)
Supplement: Supplementary Table S1 [file BSR-2020-3639_supp.pdf]

Supplementary Table 1 Primers used for RT-qPCR

**Gene**	**Sequence**	
TLR4	F	AGACCTGTCCCTGAACCCTAT
	R	CGATGGACTTCTAAACCAGCCA
GAPDH	F	GGAGCGAGATCCCTCCAAAAT
	R	GGCTGTTGTCATACTTCTCATGG
HuR	F	GGGTGACATCGGGAGAACG
	R	CTGAACAGGCTTCGTAACTCAT
PPARγ	F	GGGATCAGCTCCGTGGATCT
	R	TGCACTTTGGTACTCTTGAAGTT
C/EBPa	F	TTGTGCCTTGGAAATGCAAAC
	R	TCGGGAAGGAGGCAGGAAAC
IL1β	F	ATGATGGCTTATTACAGTGGCAA
	R	GTCGGAGATTCGTAGCTGGA
IL-6	F	ACTCACCTCTTCAGAACGAATTG
	R	CCATCTTTGGAAGGTTCAGGTTG
